# Different Evaluations Exist between Men with Erectile Dysfunction and Their Female Partners When Using Erectile Hardness Model: An Interesting, Observational, and Cross-Sectional Field Survey

**DOI:** 10.1155/2020/2302348

**Published:** 2020-02-10

**Authors:** Jingjing Gao, Yuyang Zhang, Hu Li, Pan Gao, Xiansheng Zhang

**Affiliations:** The First Affiliated Hospital of Anhui Medical University, Hefei, Anhui Province, China

## Abstract

It is an interesting clinical phenomenon that when evaluating the erectile function of men with erectile dysfunction by couples, respectively, using the erectile hardness model, there will exist the evaluation difference between men and their female partners. This phenomenon reflects the problem of communication and cognition between husband and wife in ED patients. To explore the influencing factors associated with this clinical phenomenon, we conducted this interesting, observational, and cross-sectional field survey. We enrolled 385 couples from the andrology clinics of the first affiliated hospital of Anhui Medical University from December 2017 to December 2018. The demographic data of couples, the medical history, sexuality and the characteristics of ED, and anxiety and depression of the couples were collected through face-to-face interview and questionnaires. The couples were divided into two groups containing 238 couples and 147 couples, respectively. We divided couples into difference group including couples which have inconsistent evaluation results from touching the erectile hardness model and no difference group including couples which have consistent evaluation results from touching the erectile hardness model, respectively. The difference group where the couples share different evaluation results reported higher erectile hardness grade from men than from their female partners (male > female: 73.11% *vs*. male < female: 26.89%). The scores of IIEF-5 in difference group and no difference group are 13.43 ± 5.75 and 16.82 ± 8.23, respectively. The average grades evaluated from men and women in difference group are 2.79 ± 0.85 and 2.45 ± 0.63, respectively. The average grades evaluated from couples in no difference group are 3.02 ± 0.45. Through statistical comparison and logistic regression analysis, duration of ED > 16 months, seeking treatment from female, negative communication state, and depression from men are the relevant factors accounting for the different evaluation results. This phenomenon reflects the problem of communication and cognition between husband and wife in ED patients. As for couples with these risk factors, we cannot focus only on the oral medication which only restores the penile erectile function. More importantly, we must combine the sexual counseling and sexual knowledge education with the drug treatment. When the two treatments are tightly integrated, not only the penile erection but also the gap of couples can be restored which is the best result of the ED treatment.

## 1. Introduction

Erectile dysfunction (ED), defined as the persistent inability to attain and/or maintain an erection sufficient for sexual performance for at least six months, is one of the most common diseases in males [1]. ED is a complicated interaction between the etiology of vascular, neurogenic, hormonal, psychogenic, iatrogenic, and anatomic causes, which plays an important role in the occurrence of ED [2].

Several large epidemiological studies have shown a high prevalence and incidence of ED worldwide. In the Men's Attitude to Life Events and Sexuality Study, which included 20 to 75-year-old men from 8 countries (United States, United Kingdom, Germany, France, Italy, Spain, Mexico, and Brazil), the ED prevalence, assessed by International Index of Erectile Function (IIEF), ranged from 22% in the United States to 10% in Spain [3]. In a study surveying the prevalence of ED among type 2 diabetic Chinese men, among subjects with ED, the most prevalent was mild ED (28.9%), followed by mild-to-moderate (27.9%), moderate (13.4%), and severe (9%) ED [4]. A project launched for estimating the likely worldwide increase in the prevalence of ED in the next 25 years projected that ED will affect 322 million by 2025 [5]. It is evident that ED has become a measurable health disorder for men globally that requires medical and public health attention.

ED has biological, psychological, and social effects on the patients and their sexual partners [6]. A study conducted in China concluded that the prevalence of anxiety and depression were 79.82% and 79.56% in Chinese ED patients and the prevalence and severities of anxiety and depression increased as the ED severity increased [7]. The effects of ED on the partners are strikingly similar to the effects on the patient. When erectile dysfunction occurs in a man, his female partner will suspect her attractiveness and worry that he is potent with other people. These anxious thoughts influence their confidence and lead to anxiety and depression [6]. In conclusion, ED can cause frustration, anxiety, and depression for couples, potentially resulting in separation and/or divorce with the progress of illness. The vicious cycle of anxiety and erectile dysfunction encompasses the entire relationship between the patient and the partner. With the development of this vicious circle, the couples will decrease the frequency of intercourse, time together, and communication [8]. In addition, the Female Experience of Men's Attitudes to Life Events and Sexuality study showed that women engaged less frequently in sexual activity after their partner developed ED and that their sex life was less satisfactory when the ED of their partner was severe. Similar results had been reported by other authors [9]. A research found that compared to the general population, the quality of life in people with ED was known to be decreased to on average 10% [10]. It concluded that ED not only harms the health of men but also damages the harmonious relationship between couples.

The emergence of phosphodiesterase type 5 inhibitor in 1998 dramatically altered the treatment landscape for erectile dysfunction [11]. This targeted treatment is convenient for patients and physicians. The clinical efficacy of nonselective treatment for ED can reach 60%–80% [12]. On the contrary, high rates of treatment discontinuation were present in several studies, ranging from 14% to 57% [13–16]. Higher PDE5 discontinuation rates were found in other studies, reaching 80.4% [17]. It is clear that there is a significant disparity between efficacy and continuation rates. Exploring this “disparity phenomenon,” we hypothesized that sexual dysfunction typically involves both physiological and psychological aspects, and such medications, although they improve penile neurovascular response, do not address the complex psychological and relationship issues that often accompany a sexual problem. Without exploring the relational issues that result from ED, the treatment efficacy would be limited.

In our daily male outpatient work, we found an interesting phenomenon: when using the erectile hardness measurement model for evaluating and comparing the erectile function of the men in the past six months, the couple who came to the male outpatient for ED came to a different conclusion. More often, the women's response to erectile hardness is more objective and real than the patient himself. This phenomenon reflects the problem of communication and cognition between husband and wife in ED patients. In the general male population, the prevalence of ED has increased to approximately 20%, but less than 30% of patients seek treatment [18]. Due to factors such as Chinese traditional culture, cognitional differences of the patients and their spouses, most men often show sorrow and anxiety about the disease and misconceive this disease. This makes the male patients in the face of the doctor only emphasize the organic factors of their erectile dysfunction, avoiding the related effects of the disease on the sexual partner and both sides. This will cause the doctor to ignore the effect of the ED on the patient's relationship, and the treatment to the patient's erectile function is limited to the use of drugs. However, except in ideal circumstances when these psychosocial forces are not present, dispensing a tablet to reverse these powerful forces is not likely to succeed [19]. Consequently, the exploration of the influencing factor of the aforementioned details will be helpful to the exploration of the psychological factors owing to the illness itself and to attach sexual counseling and sexual education to the drug therapy to improving the treatment efficacy of ED.

The purpose of this paper is to explore the factors influencing the differences in the evaluation of the penile hardness model between husband and wife. We explore relevant factors from multiple perspectives including duration of ED, duration of relationship, frequency of sexual intercourse, the main reason for treatment of ED, the state of communication, and the psychological burdens of the couples. Moreover, we want to inform andrologists that when treating ED patients with such risk factors, combined drug therapy, sexual counseling, and sex education will achieve better therapeutic goals.

## 2. Methods

### 2.1. Patient Selection

Patients who were referred to the Department of Andrology, the First Affiliated Hospital of Anhui Medical University (Hefei, China), for the erectile dysfunction from December 2017 to December 2018, were enrolled in this study. This study was reviewed and approved by the Anhui Medical University Research Subject Review Board. Informed consent was obtained from all patients before study. To be enrolled in the study, all subjects had to meet the following criteria: (a) males and their female partner aged ≥18 years; (b) the couples comprehend and speak Chinese; and (c) males having ED for more than six months with a regular heterosexual relation (at least once per week). Exclusion criteria were as follows: take medicine that could affect erectile function, the presence of a severe psychopathological disorder, and suffering from premature ejaculation (according to ISSM definition of PE). Subjects' medical and sexual histories were carefully evaluated by an experienced clinician.

### 2.2. Study Design

**Figure 1:**
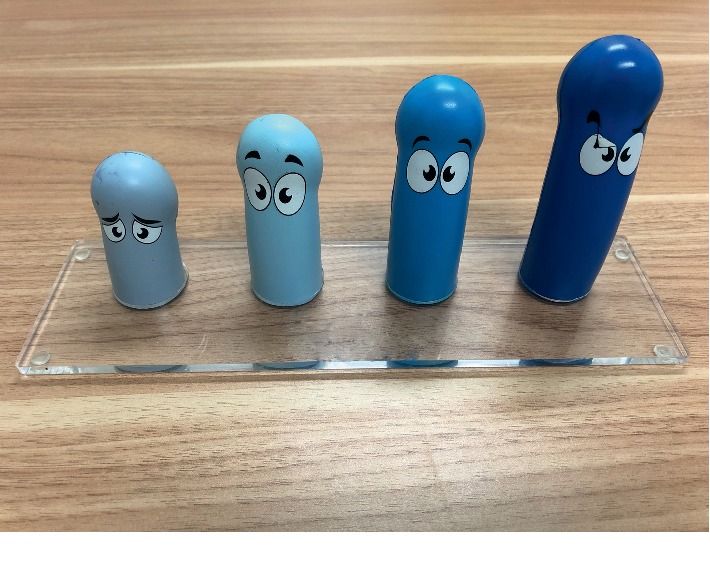
Evaluation model of erectile hardness (made by the Pfizer Inc.).

Before the official investigation begins, a presurvey was given to a small sample (*n* = 30) to modify the originally designed items to ensure that the questionnaire was comprehensive and easily understood owing to several subjective and sensitive personal questions included in the study. This survey was conducted with three steps. Firstly, a question was asked to men with ED (diagnosed with IIEF-5) and their female partner, such as “based on the previous six months, which one of the models was similar to you or your partner erectile hardness.” Then, they answered the question by the evaluation model of erectile hardness (see Figure 1). This model made by the Pfizer Inc. (Pfizer Inc., New York, NY) was the visual and tactile version of the standardized Erectile Hardness Score (EHS) tool [20]. It was originally validated and standardized in order to evaluate the efficacy of sildenafil citrate in recovering EF [21]. Its four grades represent four states of the penile, respectively, when stimulated by the sex. The dark blue penis model of the tool (score 4 at the EHS) mirrors the sentence “penis completely hard and fully rigid.” The blue penis model of the tool (score 3 at the EHS), in turn, mirrors the sentence “penis hard enough for penetration but not completely hard.” The light blue penis model of the tool (score 2 at the EHS) mirrors the sentence “penis is hard but not hard for penetration.” The light gray penis model of the tool (score 1 at the EHS) mirrors the sentence “penis is large but not hard.” Secondly, a face to face interview was conducted to collect a detailed medical history of the patients, including the duration of relationship, the cause of disease, the duration of disease, the frequency of sexual intercourse, the main reason of treatment, and the use of erectile-related drugs. Additionally, the state of couple communication includes active communication behavior and negative communication behavior [22]. Thirdly, we make two questionnaires intended for men and women to collect some information. Here, a detailed interpretation of the questionnaires follows. The first part of the two questionnaires is the same, mainly including some demographic characteristics: age, BMI, life style (smoking status and exercise status), characters, educational status, occupational status, and residence. The NEO-PI-R was used to assess the personality of the couples [23]. The second part of the questionnaire intended for men is the 5 items of International Index for Erectile Function used to measure the sexual dysfunction of the men [24]. The third part of the questionnaire attended for the couples contains the Zung self-rating anxiety/depression scales [25, 26]. The reliability of these instruments (NEO-PI-R, the Zung self-rating anxiety/depression scales, and IIEF-5) was assessed with Cronbach's alpha coefficient. The internal consistencies of the NEO-PI-R, the Zung self-rating anxiety/depression scales, and IIEF-5 were 0.84, 0.80, 0.81, and 0.79, respectively.

According to NEO-PI-R, the personality was divided into introverts and extroverts [27, 28]. Anxiety and depression, as the two indices reflecting the degree of negative psychological impact, were assessed by the Zung self-rating anxiety/depression scales, in their Chinese version [29]. Each questionnaire contained 20 questions. After the questionnaire was completed, the total scores for the Zung self-rating anxiety/depression scales were combined, divided by 80, and then compared with a standard cutoff score for anxiety or depression. A standard cutoff of 0.5 was employed such that a score <0.5 indicated no anxiety/depression and a score _>0.5 confirmed anxiety/depression. The erectile dysfunction was measured by the Chinese version of IIEF-5 [30], which is a validated five-item version of the 15-item IIEF questionnaire. It contains five questions, each of which is graded on a scale from 0 to 5 points. An IIEF-5 score >22 indicated normal erectile function and <22 indicated ED.

### 2.3. Main Outcome Measures

The main outcome was collected through combining face-to-face interview with the questionnaires. First, demographic data of couples included age, BMI, life style (smoking status and exercise status), characters, educational status, occupational status, and residence. Second, the medical history and sexuality mainly contained the reason of treatment and the frequency of sexual intercourse. Third, the characteristics of ED included the duration of ED, the scores of IIEF-5, and the grades of EHS model evaluated by couples. Moreover, anxiety and depression of the couples were assessed by the Zung self-rating anxiety/depression scales, respectively.

### 2.4. Statistical Analysis

Data analyses were carried out with SPSS version 13.0 software (SPSS Inc., Chicago, IL, USA). Descriptive statistics were used to summarize the characteristics of the subjects. The descriptive data of subjects were expressed as the mean ± standard deviation or number (percentage) when appropriate. The independent *t*-test and chi-square test were used for intergroup comparisons. Multiple logistic regression analysis was used to evaluate the association between the factors of the couples and the different evaluation results of erectile hardness model. Odds ratios and 95% CIs were calculated to examine the strength of association. For all tests, *P* value less than 0.05 was considered statistically significant.

## 3. Results

### 3.1. Characterization of the Two Groups

According to the evaluation results evaluated by couples through the erectile hardness model, we divided patients into difference group and no difference group, respectively. The difference group (GROUP 1) included couples which have inconsistent evaluation results from touching the erectile hardness model. On the contrary, the no difference group (GROUP 2) included couples which have consistent evaluation results from touching the erectile hardness model.

### 3.2. Baseline Characteristics

Overall, a total of 385 couples are enrolled in our study. They all meet our inclusion criteria and are willing to participate in our research. According to the information completed by the study couples, two groups are generated. Of the total sample, 62% (238/385) of the sample were divided into difference group (GROUP 1) and 38% (147/385) of the sample were divided into no difference group (GROUP 2).

The demographic characteristics including age, BMI, lifestyle, characters, educational status, occupational status, and residence of the patients and the corresponding female partners are shown in Table 1. The *t* test of two independent samples and the chi-square test were used for intergroup comparisons of the characteristics of the men and women, respectively. There was no statistical significance of the two groups no matter men or women.

### 3.3. Couples of the Difference Group

As shown in Table 2, the difference group not only consisted more couples but also report higher erectile hardness grade compared to the grade of their female partners (male > female: 147 (73.11%) *vs*. male < female: 64 (26.89%)).

### 3.4. Factors Associated with Different Erectile Hardness Evaluation in Couples with ED

As shown in Table 3, we comparatively analyzed the couples' sexual histories, the duration of ED, the frequency of sexual intercourse, communication state, and psychological burden. The significant differences were shown between difference group and no difference group with respect to duration of ED, frequency of sexual intercourse, the reasons of seeking treatment, communication state of the couples, and several psychological burdens from men and women.

As shown in Table 4, a logistic regression was conducted to assess whether the factors mentioned above could predict the evaluation difference. Through further correlation analysis, duration of ED > 16 months, seeking treatment from female, negative communication state, and depression from men were the factors associated with difference erectile hardness evaluation between couples.

### 3.5. Erectile Function of the Men in Two Groups

As shown in Table 5, the erectile function of the men was evaluated through the IIEF-5 by men and the erectile hardness model by the men and women. The scores of IIEF-5 in difference group and no difference group are 13.43 ± 5.75 and 16.82 ± 8.23, respectively. The average grades evaluated from men and women in difference group are 2.79 ± 0.85 and 2.45 ± 0.63, respectively. When compared with the no difference group, the men from difference group scored lower through IIEF-5. The results are the same as the method of the erectile hardness model. As mentioned above, the men from difference group scored lower through the erectile hardness model compared to their female partner.

## 4. Discussion

ED is a disease in which multiple factors are involved in the occurrence and the progression of it, and its etiology is complicated [31]. Among these factors, both husband and wife play an important role. In our clinical work, we find that as for men with erectile dysfunction, we need to not only evaluate the erectile function of the men and the related risk factors but also fully master how the relationship between husband and wife goes. Answers of these questions allow us to fully understand sexual cognition and the state of sexual communication of the two sides. When we treat men with ED only through drugs, we cannot restore these risk factors existing between couples which play important roles in the progress of the disease.

In our outpatient visit, we found that the husband and wife diverged on the evaluation of the male erectile function by the erectile function model when the couple visits us for ED together. The differences in sexual cognition and the lack of communication on sexual topics of couples were revealed through this simple phenomenon. This study was conducted to further explore the relevant factors affecting the evaluation results of couples. We concluded the factors that are closely related to the differences between the evaluation results of couples including the course of ED, the cause of treatment of ED patients, the communication status of couples, and the depression of men.

In this study, male patients in the difference group were reported a longer course of ED than the no difference group. ED has a physical, mental, and social impact on the quality of life of patients and their partners [6]. We think that the communication barrier between couples is prolonged as the duration of ED. For men, ED patients often show anxiety and depressive symptoms related to sexual performance. For women, this mental stress cannot be ignored. Changes in male sexual behavior can confuse his sexual partners and even make her generate some strange ideas, such as their spouse is losing interest with her [32]. The mental impact of ED on both men and women will gradually increase as the disease progresses, which will gradually undermine the sexual communication between the husband and wife. For women, male with ED also has a series of adverse effects on her sexual functions including sexual desire disorder, sexual excitement, orgasm disorder, and pain in sexual intercourse. A cross-sectional study conducted in Taiwan showed that women with ED partners had lower scores in FSFI's total score and field scores compared with women without ED partners [33]. Women continue to reduce sexual contact with patients because of these bad effects. Studies have also shown that when male patients developed ED, they also reduce the sexual life frequency of sexual partner to avoid embarrassing situation [34]. The decline in sexual frequency will get worse as the duration of ED prolongs. It will reduce the quality of sexual life of both parties, reduce communication between husband and wife, and undermine the harmonious relationship between husband and wife.

For men, they are more likely to refuse treatment than women for most ailments [35]. In China, influenced by traditional culture of conservative attitude towards sex, men are even more reluctant to admit their decline in sexual function when faced with sex-related issues such as ED. A clinical-based survey in China named Help-seeking behavior for erectile dysfunction found that many patients were clinically diagnosed with ED and might not realize that they had erectile problems at first [36]. For women, they are always able to feel small changes in sexual behavior during sexual intercourse earlier than men. Facing the embarrassed question of ED, the husband and wife have adopted two very different attitudes. This very different attitude led to assessment differences in the face of the evaluation model. This conclusion agrees with our findings. These different attitudes of couples in the face of sexual problems will affect the treatment of ED. This also reminds us that in the process of ED treatment, sexual knowledge education and consultation for couples which are lacking in different groups are of great significance.

Our research indicates that the state of communication between husband and wife affects the judgment on the penile hardness model. The ED problem is not only a male problem, but also a problem for couples. Culture defines the role of men and women, how they relate to each other, their cultural group, and the community [37]. During the diagnosis and treatment of ED, women play an important role in the provision of patient medical history and patient compliance. The women's understanding comes from the good communication between husband and wife and the correct understanding of sexual knowledge. Therefore, when the two sides communicate negatively, it will result in great resistance to the diagnosis and treatment of ED. In a study on the cessation of ED treatment, 9.3% of men pointed out that the relationship between husband and wife is the main reason for stopping drug treatment [38]. So, when there is negative communication between the couple, this will inevitably lead to errors in their knowledge of the disease. When facing the hardness evaluation model, this error will be magnified. The phenomenon also reminds us that when treating ED patients, in addition to the use of drugs, we must deal with the communication state of the couples.

Mental factors are closely related to ED. In the United States, mental problems or stress are important predictors of ED and the OR is 3.6 [39]. Anxiety and depression are both risk factors for ED and important neurological effects of ED on men. ED and depression are considered to be two-way effects, and the two conditions reinforce each other [40]. An estimated 25% of men with depressive symptoms may suffer from ED [41, 42]. Gradually, psychological disorders grew as the ED duration progressed. Not only the incidences but also the severities of anxiety and depression significantly rose with the duration of ED. Depression can reduce libido and aggravate the disease of ED [43]. In the meantime, unsatisfied sexual life can aggravate depression. When men are suffering from depression due to ED, their attitude toward ED is more evasive. The evasive attitude will be uncovered when faced with a penile erection model.

Our research inspiration is from a small phenomenon found in Andrology outpatient work: different results between couples in using models to assess male erectile hardness grade. This small difference seems to have nothing to do with the treatment of ED. In fact, through our research, we find that there are many deep problems hidden under this phenomenon. Through in-depth analysis, we summarize four factors that influence the outcome, the duration of the ED, the reasons for the visit, the state of communication between the spouses, and the mental factors. These related factors also suggest that ED destroys not only the male penile erection function but also the relationship between husband and wife. The drug only treats the erectile function of the male, and the better treatment is to restore the harmonious sexual life of the patient and his wife. In response to these factors, sexual counseling and sexual education and drug therapy can be combined to achieve better therapeutic results.

There are some limitations in our article. (a) We performed the correlation research on the questionnaires of erectile dysfunction (IIEF-5) and the EHS from men and women. (b) This study is a cross-sectional study. Later, we will conduct a cohort study to further follow up patients in different groups to compare the response and continuation to PDE5i. (c) At the same time, we also conduct further research on the sexual function of the female partners using the Female Sexual Function Index. (d) In the later stage, we will carry out relevant sexual knowledge education and sexual counseling and apply the research results to the treatment of clinical ED.

## 5. Conclusion

ED not only affects the male penile erectile function but also destroy the sexual relationship and communication on sexual topics between couples. In our study, duration of ED > 16 months, seeking treatment from females and negative communication state and negatively psychological burden (depression) from men were the pivotal factors for different judgment grades between the spouses. As for couples with these risk factors, we cannot focus only on the oral medication which only restores the penile erectile function. More importantly, we must combine the sexual counseling and sexual knowledge education with the drug treatment. When the two treatments are tightly integrated, not only the penile erection but also the gap of couples can be restored which is the best result of the ED treatment.

## Figures and Tables

**Table 1:** Demographic characteristics of couples with ED complaint in difference and no difference groups.

	Difference group (*n* = 238)				No difference group (*n* = 147)			
	Man		Female partner		Man		Female partner	
Age (years)	38.29 ± 10.73		36.37 ± 9.25		37.80 ± 9.47		34.55 ± 11.74	
BMI (kg/m2)	24.58 ± 2.25		22.09 ± 4.33		23.67 ± 3.06		22.21 ± 3.79	
Lifestyle (*n*%)								
Smoking	154	64.71%	10	4.20%	90	61.22%	11	7.48%
Exercise	85	35.71%	47	19.75%	45	30.61%	48	32.65%
Characters (*n*%)								
Introversion	165	69.33%	146	61.34%	74	50.34%	70	47.62%
Extroversion	73	30.67%	92	38.66%	73	49.66%	77	52.38%
Educational status (*n*%)								
High school or less	143	60.08%	180	75.63%	58.50%	90	61.22%	58.50%
University graduate	95	39.92%	58	24.37%	41.50%	57	38.78%	41.50%
Occupational status (*n*%)								
Student	66	27.73%	116	48.74%	27.89%	33	22.45%	27.89%
Unemployed	139	58.40%	103	43.28%	61.90%	96	65.31%	61.90%
Employed	33	13.87%	19	7.98%	10.20%	18	12.24%	10.20%
Resident (*n*%)								
Urban	94	39.50%	145	60.92%	52	35.37%	44	29.93%
Rural	144	60.50%	93	39.08%	95	64.63%	103	70.07%

ED = erectile dysfunction; difference group = group including couples with different evaluation results of the erectile hardness model; no difference group = group including couples with no different evaluation results of the erectile hardness model; BMI = body mass index.

**Table 2:** Comparison of erectile hardness evaluated by the erectile function model from men and female partner.

		*N*	% for their groups	% for all subjects
Difference group	Evaluation grade male > female	174	73.11	45.19
	Evaluation grade male < female	64	26.89	16.62

No difference group	Evaluation grade male = female	147	100	38.18

Difference group = group including couples with different evaluation results of the erectile hardness model; no difference group = group including couples with no different evaluation results of the erectile hardness model; evaluation grade = grade evaluated by men and women, respectively, through the erectile hardness model.

**Table 3:** Associated factors for different erectile hardness evaluation in couples with ED.

	Different group (*n* = 238)		No different group (*n* = 147)		*P* value
Duration of ED (months)	16.25 ± 10.74		13.25 ± 9.84		<0.001
Duration of relationships (years)	9.27 ± 4.12		9.05 ± 4.23		^*∗*^
Frequency of sexual intercourse (times)	3.29 ± 2.25		5.77 ± 3.13		<0.001
The main reason for treatment (%)					<0.001
From male	106	44.54%	96	65.31%	^*∗*^
From female partner	132	55.46%	51	34.69%	^*∗*^
The state of couple communication (%)					<0.001
Active	92	38.66%	77	52.38%	^*∗*^
Negative	146	61.34%	70	47.62%	^*∗*^
Psychological burden (%)					^*∗*^
Anxiety from men	43	18.07%	19	12.93%	<0.001
Anxiety from female partner	23	9.66%	13	8.84%	^*∗*^
Depression from men	30	12.61%	11	7.48%	<0.001
Depression from female partner	13	5.46%	8	5.44%	^*∗*^

*Note*. Values were considered statistically significant when *P* < 0.05; statistical method: ANOVA—analysis of variance; *χ*^2^—chi-square test; ^*∗*^*P* > 0.05.

**Table 4:** Multiple logistic regression analysis of risk factors for different erectile hardness evaluations in couples with ED.

	OR	95% CI	*P* value
Duration of ED > 16 months	3.35	1.56∼7.72	<0.001
The main reason for treatment from female partner	2.18	1.96∼5.23	<0.001
Negative state of couple communication	3.02	2.11∼6.94	<0.001
Depression from men	2.07	1.15∼4.29	<0.001

ED = erectile dysfunction, OR = odds ratio, CI = confidence interval.

**Table 5:** Erectile function of the men in two groups evaluated by IIEF-5 and Erectile Hardness Model.

	Difference group (*n* = 238)		No difference group (*n* = 147)	
	Men	Female partner	Men	Female partner
IIEF-5	13.43 ± 5.75^*∗*^	—	16.82 ± 8.23	—
EHS	2.79 ± 0.85^*∗*^^#^	2.45 ± 0.63	3.02 ± 0.45	3.02 ± 0.45

^*∗*^Compared with the no difference group, significant differences were found in the difference group. ^#^Compared with female partner, significant differences were found in men's difference group. Difference group = group including couples with different evaluation results of the erectile hardness model; no difference group = group including couples with no different evaluation results of the erectile hardness model; IIEF-5: the 5-item version of the International Index of Erectile Function; EHS: grade evaluated by the model of erectile hardness.

## Data Availability

Datasets supporting the conclusions of this article are available and can be requested from the corresponding author.
